# Joint interactions of carbon and nitrogen metabolism dominated by bicarbonate and nitrogen in *Orychophragmus violaceus* and *Brassica napus* under simulated karst habitats

**DOI:** 10.1186/s12870-022-03646-1

**Published:** 2022-05-26

**Authors:** Antong Xia, Yanyou Wu

**Affiliations:** 1 Research Center for Environmental Bio-Science and Technology, State Key Laboratory of Environmental Geochemistry, Institute of Geochemistry, Chinese Academy of Sciences, 550081 Guiyang, P.R. China; 2 University of Chinese Academy of Sciences, Beijing, 100049 People’s Republic of China

**Keywords:** Bicarbonate, Carbon/nitrogen metabolism, Nitrogen source, Photosynthesis, Karst adaption

## Abstract

Karst habitats are uniquely characterized by high bicarbonate, high nitrate, and low ammonium, which are in-conducive to their growth and biodiversity. The occurrence of inorganic carbon and nitrogen in karst soil profoundly affects the carbon/nitrogen metabolism and adaptability of plants. However, there has been no final conclusion to the joint interactions of carbon and nitrogen metabolism in plants under karst habitats. In this study, we selected a karst-adaptable plant *Orychophragmus violaceus* (*Ov),* and a non-karst-adaptable plant *Brassica napus* (*Bn*) as experimental plants, and compared their joint effects of carbon and nitrogen metabolism under simulated karst habitats. It was found that the two species had different joint effects of carbon and nitrogen metabolisms. Bicarbonate and nitrate joint promoted photosynthetic activity and glucose metabolism, facilitating the carbon/nitrogen metabolism and growth of *Ov*, but their impacts on the carbon and nitrogen metabolism were insignificant in *Bn.* Bicarbonate and ammonium joint inhibited the photosynthesis and nitrogen metabolism, but promoted water use efficiency in *Ov*, leading to its enhance of growth reduction, ammonium toxicity alleviation, and drought resistance*,* while they inhibited the water use efficiency of *Bn.* In general, bicarbonate and nitrate/ammonium more significantly joint affected the carbon and nitrogen metabolism in *Ov* than *Bn*, which is vital for *Ov* to adapt to karst habitats.

**Supplementary Information:**

The online version contains supplementary material available at 10.1186/s12870-022-03646-1.

## Background

Karst habitats, featuring drought, high bicarbonate, high nitrate and low ammonium, can cause ecological degradation, which has attracted widespread attention [1]. The formation of karst habitats is complex. On the one hand, the karst process promotes the formation of skylights and caves, which are in-conducive to water storage and thus lead to a karst drought adversity [2]. On the other hand, soils formed by carbonate contribute to the abundance of bicarbonate, consuming lots of H^+^ and thus creating a high pH habitat. Besides, ammonium in such habitats will change into ammonia through volatilization due to its high pH, resulting in a nitrate-abundant and ammonium-low environment [3]. Most plant species are non-adaptable to a karst habitat because their photosynthesis will be inhibited due to disrupted water balance and conductance [4, 5], while a few are well adaptable to it thanks to their unique strategies of inorganic carbon and nitrogen metabolism.

In addition to atmospheric carbon dioxide, dissolved inorganic carbon (DIC) existing in carbon dioxide (CO_2_) and bicarbonate (HCO_3_^−^) was demonstrated to be able to participate in the carbon assimilation. The pH value determines the carbon forms, and as the pH increase, DIC in water medium is mainly in the form of HCO_3_^−^, which are used by algae and plants, such as *Orychophragmus violaceus* [1], *Chlamydomonas reinhardtii* [6], *Camptotheca acuminata* [7], *Potamogeton crispus* and *Potamogeton malaianus* [8, 9]. In a karst habitat, carbon dioxide and bicarbonate are alternatively used by plants and the latter can promote carbon assimilation and improve water availability as well as glucose metabolism [10]. Carbonic anhydrase (CA) catalyzes HCO_3_^−^ to CO_2_ and H_2_O, contributing to maintaining photosynthesis [1, 2]. Furthermore, bicarbonate is involved in total inorganic carbon assimilation, and contributes 9.71% to the total inorganic carbon assimilation in *Camptotheca acuminate* under simulated karst drought [7]. It can also adjust the glucose metabolism in plants. Yao [10] indicated that 3 mM bicarbonate increased both the glycolytic pathway (EMP) and the pentose phosphate pathway (PPP) of *Broussonetia papyrifera* (*Bp*), resulting in the enhancement of the total glucose metabolism. Nitrate and ammonium are the primary inorganic nitrogen utilized by plants, and the former is the primary nitrogen source for most plants under aerobic soil conditions [11, 12]. Typically, nitrate also plays an important role in physiological processes [13], but energy and NADPH/NADH reducing agents are required for plants to uptake it. Compared to ammonium, nitrate is theoretically more accessible for plants, but nitrate reduction requires 15 ATP, whereas ammonium assimilation requires only 5 ATP [14]. However, excessive ammonium leads to intracellular acidification and vein damage, which will result in the decline of photosynthetic products and plant growth [15]. In general, both nitrate nitrogen and ammonium nitrogen are necessary for the growth of most plants, and their distribution in soil determines the nitrogen utilization characteristics of plants [16–18]. Therefore, it is crucial to figure out the effects of nitrate and ammonium on the nitrogen utilization characteristics in plants. A karst habitat features nitrate-abundant and ammonium-rare soil, where karst-adaptable plants can better survive than non-karst-adaptable plants [1]. Lu [19] indicated that *Ov* would have higher nitrate utilization than *Bn* under simulated karst habitats. While these studies describe the utilization of inorganic carbon or nitrogen by plants in karst habitats, the symbiotic metabolism of these two elements remains unclear.

The carbon and nitrogen metabolisms of plants are closely coupled by energy and carbon skeletons. On the one hand, carbon assimilation affects nitrate reduction through the energy produced by photosynthesis, while the glycolytic pathway (EMP) supplies ATP to the nitrogen metabolism and recycles 75%of the cycle [20]. On the other hand, one ammonium ion released in mitochondria during serine biosynthesis from two glycines is re-assimilated in chloroplast by reduced ferridoxin. Meanwhile, nitrogen reduction not only provides enzymes and protein for photothesis, EMP and PPP, which are vital carbon skeletons of carbon metabolism, but also offers nitrogen to the composition of chlorophyll [17] as well as NAPDH to the pentose phosphate pathway (PPP) [18]. The carbon metabolism includes photosynthesis, EMP, PPP, and enzymes such as Ribulose bisphosphate carboxylase oxygenase (Rubisco) and sucrose synthetase (SS) [21–23]. In contrast, the nitrogen metabolism contains nitrate reduction and ammonium assimilation, which are obviously affected by nitrate reductase (NR) and glutamate synthase (GOGAT) [24]. The above indicators determine the carbon and nitrogen growth in plants regarding biomass and accumulation. Consequently, the above coupling relationships were determined to clarify the joint effects of carbon and nitrogen metabolisms in plants.

In a karst habitat, bicarbonate, nitrate, and ammonium have a profound influence on the inorganic carbon and nitrogen metabolisms [1, 2], and therefore it is essential to clarify the conjugation of bicarbonate, nitrate, and ammonium to carbon and nitrogen metabolism in plants. However, the growth in plants is influenced by multiple factors, such as climate, rainfall, and biodiversity, making it difficult to quantify the conjugation of carbon and nitrogen metabolisms [3, 19]. In this study, we simulated karst habitats in an artificial greenhouse, and then cultivated the *Orychophragmus violaceus* (*Ov*, karst-adaptable plant) and *Brassica napus* (*Bn*, non-karst-adaptable plant) to determine the joint effects of carbon and nitrogen metabolisms on different plant species. The followings are the main objectives of this study: (1) to compare the differential joint effects on the carbon and nitrogen metabolisms of bicarbonate and nitrate/ammonium in different plant species under the environment of a simulated karst habitat; (2) to determine the karst adapting mechanisms of carbon and nitrogen in karst-adaptable plants.

## Methods

### Plant materials

The experiments were carried out in an artificial greenhouse at the Institute of Geochemistry, Chinese Academy of Sciences (Guiyang, China) with a dimension of 10 (L) × 5 (W) × 4 (H) m. The light was provided by metal halide lamps (HPI-T400W/645, Philips, the Netherlands) while the temperature was controlled by air conditioning. Seedlings were then incubated in a photoperiod of 16/8 h under light/dark conditions, along with 500 ± 23 μmol m^− 2^ s^− 1^ of photosynthetic photon flux density. The greenhouse conditions were set as:temperature (day/night): 25/19 °C, constant light time of 12 h per day; relative humidity range: 55–60%. In this study, we selected *Ov* (karst-adaptable plant) and *Bn* (non-karst adaptable plant) as experimental plants and disinfected the seeds with 70% ethanol for 1 min with constant agitation. Additionally, the seeds were repeatedly rinsed 3–5 times and soaked for 6–8 h. The seeds were sown in 12-hole trays (size: 19 × 15 × 9.5 cm) and grown on a substrate (perlite: vermiculite = 1:3) with the modified Hoagland solution [2], which was changed every 3 days. After 28 days, the seedlings were transplanted and followed by 48 h without nitrogen. Finally, we chose 48 plants with uniform growth of *Ov* and *Bn* and randomly divided them into 16 groups (3 plants each group), so as to the following stress treatments.

### Stress treatments

The continuous rainfall cycle in karst areas is usually 1–8 days, so karst drought (PEG6000, 10 g/L) was simulated for 8 days to cultivate *Bn* and *Ov* seedlings (Table 1). The bicarbonate of wet soil is more than 10 mM under karst habitats. Therefore, 10 mM of NaHCO_3_ was prepared to simulate the bicarbonate environment, and the pH was adjusted to 8.30 ± 0.05 with 1 mol/L KOH so that the bicarbonate exists in an alkaline environment [2, 5]. Quantitatively, the culture solution was changed daily at 9:00 a.m., and the soil water content was maintained at 20–25%. Equal amounts of plants were assigned to the measurement of the photosynthesis, carbon and nitrogen metabolism enzymes, leaf carbon and nitrogen contents, and biomass.

**Table 1:** The condition of simulated karst habitats

Treatment	Reagents	Substance content (mM·L^− 1^)
Control	–	–
HCO_3_^−^	NaHCO_3_	10
nitrate:ammonium	NaNO_3_/NH_4_Cl	1:9
nitrate:ammonium	NaNO_3_/NH_4_Cl	5:5
nitrate:ammonium	NaNO_3_/NH_4_Cl	9:1
Nitrogen removal nutrient solution	Hogland nutrient solution (without nitrate and ammonium)	[2]
pH	KOH	8.30 ± 0.05
Drought	PEG6000	10 g·L^−1^

### Photosynthesis

Photosynthesis was measured with a portable photosynthesis measurement system (LI-COR, Lincoln, USA), of which the parameters were manually set: light intensity: 500 μmol m^− 2^ s^− 1^ PPFD; temperature: 25 °C; CO_2_ concentration: 400 μmol m^− 2^ s^− 1^. Photosynthetic parameters include net photosynthetic rate (Pn), stomatal conductance (Cond), transpiration rate (Tr), and intercellular carbon dioxide (Ci). They were measured on the third cotyledon every 2 days, and we calculated the water use efficiency (1).

1 $$\mathrm{WUE}\;\left(\%\right)=\mathrm{Pn}/\mathrm{Tr}$$

### The carbon and nitrogen metabolism enzymes

0.1 G of *Ov* and *Bn* fresh leaves (the 3rd or 4th euphylla of the seedling) were weighed and ground with liquid nitrogen. Then, the rubisco, SS, NR, GOGAT, PFK (A_PFK_) and G6PDH activity (A_G6PDH_) were tested with the bio enzyme kit (Sangon, Shanghai, China)

The total glucose metabolism activity (EA_∑_), EMP and PPP, GC and RC_RUBP_ were calculated by the methods adopted by Yao and Wu [10].

2 $${\mathrm{EA}}_{\sum }={\mathrm{A}}_{\mathrm{PFK}}+{\mathrm{A}}_{\mathrm{G}6\mathrm{PDH}}$$

3 $${\mathrm{E}}_{\mathrm{E}\mathrm{MP}}={\mathrm{A}}_{\mathrm{PFK}}/{\mathrm{E}\mathrm{A}}_{\sum }$$

4 $${\mathrm{E}}_{\mathrm{PPP}}={\mathrm{A}}_{\mathrm{G}6\mathrm{P}\mathrm{DH}}/{\mathrm{E}\mathrm{A}}_{\sum }$$

5 $$\mathrm{GC}={\mathrm{E}}_{\mathrm{E}\mathrm{MP}}\times \mathrm{Pn}$$

6 $${\mathrm{RC}}_{\mathrm{RUBP}}={\mathrm{E}}_{\mathrm{PPP}}\times \mathrm{Pn}$$

### Leaf carbon and nitrogen content

15–25 mg of the dried *Ov* and *Bn* leaves were wrapped in the tin foil, the leaf carbon and nitrogen contents were measured with the elemental analyzer (Elementar, Germany).

### Biomass

The plants were dissected into three parts, roots, stems and leaves, then were dried at 108 °C for 30 min and 75 °C after cleaning. The samples were weighed by analytical balance (accuracy 0.0001 g) to obtain the biomass and plants and calculate the root/shoot ratio(R/S, %).

7 $$\mathrm{R}/\mathrm{S}\;\left(\%\right)={\mathrm{Dw}}_{\mathrm{root}}/\left({\mathrm{Dw}}_{\mathrm{stem}}+{\mathrm{Dw}}_{\mathrm{leaves}}\right)\ast 100\%$$

### Statistical analysis

The experimental data were processed as follows: the images were drawn with Origin 9.0; ANOVA, Duncan and LSD in SPSS 25 were used to analyze the data; and the differences between the means were considered significant when the *p*-value was less than 0.05. The data were expressed as “mean ± standard deviation”.

## Results

### Photosynthesis

**Fig. 1:**
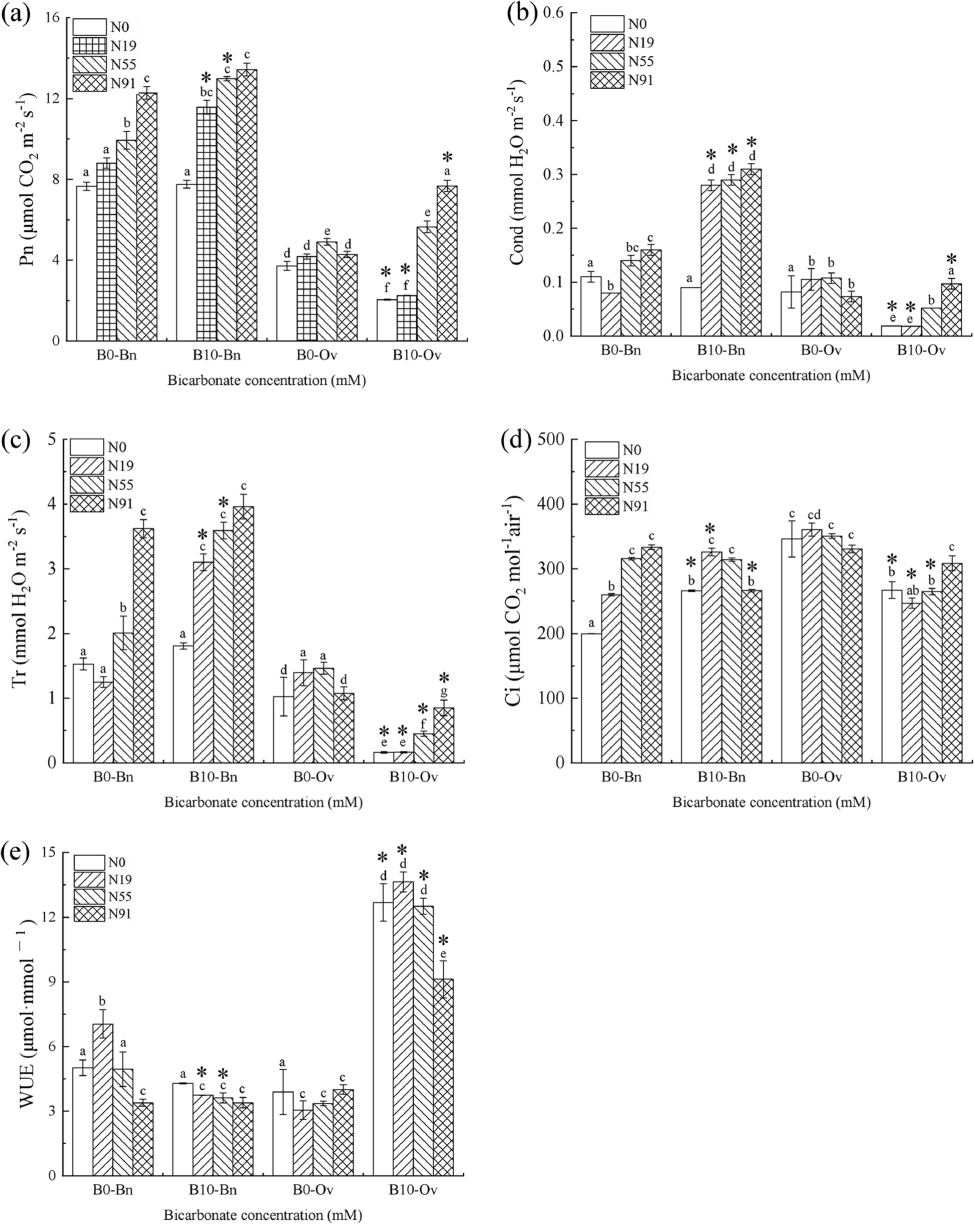
Photosynthetic characteristics of the *Bn* and *Ov* seedlings under simulated karst habitats. Pn-Photosynthetic rate, Cond-Stomatal conductivity, Tr-Transpiration, Ci-Intercellular CO_2_, WUE-water use efficiency, *Bn*-*Brassica napus*, and *Ov*-*Orychophragmus violaceus*. The bicarbonate was denoted as B0 (HCO_3_^−^: 0 mM), and B10 (HCO_3_^−^:10 mM). Nitrogen was denoted as N0(no nitrogen), N19 (nitrate:ammonium = 1 mM: 9 mM), N55 (nitrate:ammonium = 5 mM: 5 mM), and N91 (nitrate:ammonium = 9 mM: 1 mM). Each value represents the mean ± SD (*n* = 3). The mean values marked with different letters (a,b,c,d) significantly differ at *P* < 0.05

In Fig. 1, only nitrate promoted the photosynthesis of *Bn*, and Pn increased significantly at B0N91; while bicarbonate and nitrate/ammonium were not joint to promote the photosynthetic activity in *Bn* as they were insignificantly changed. Meanwhile, the Cond in *Bn* was promoted by the bicarbonate and nitrate joint and increased significantly at B10N91.

The bicarbonate and nitrate joint promoted the photosynthesis in *Ov*, of which Pn, Cond and WUE significantly increased at B10N91, while it inhibited Tr in *Ov*, which was significantly reduced at B10N91. The bicarbonate and ammonium joint only significantly promoted WUE in *Ov*, but inhibited the photosynthesis of *Ov*, resulting in a significant reduction in Pn, Cond, Tr, and Ci of *Ov* at B10N19.

### Carbon and nitrogen metabolizing enzymes

**Fig. 2:**
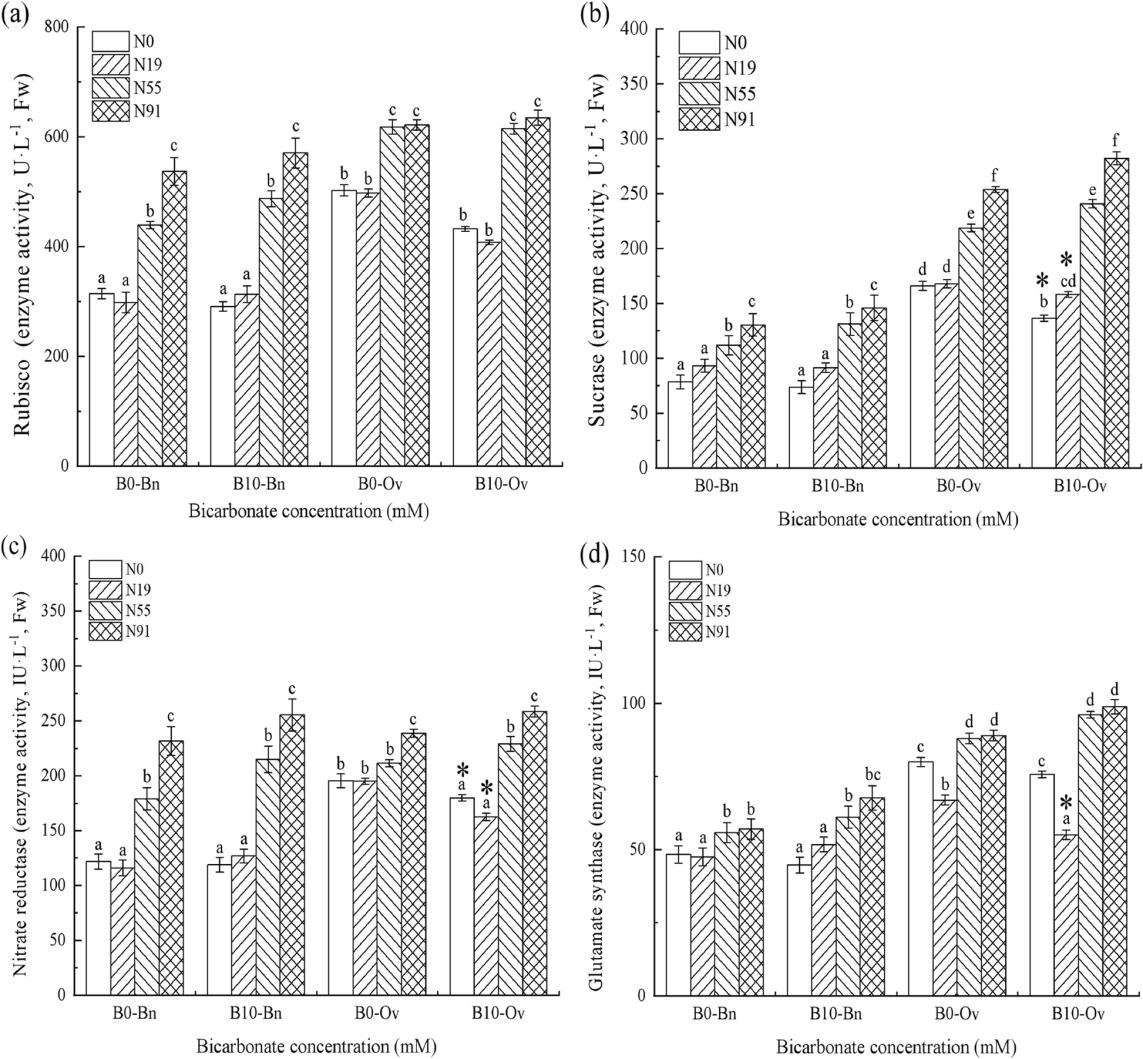
Carbon and nitrogen metabolism enzymes of the *Bn* and *Ov* seedlings under simulated karst habitats. *Bn*, *Brassica napus*; *Ov*, *Orychophragmus violaceus*. The bicarbonate was denoted as B0 (HCO_3_^−^: 0 mM), and B10 (HCO_3_^−^:10 mM), respectively. Nitrogen was denoted as N0 (no nitrogen), N19 (nitrate:ammonium = 1 mM: 9 mM), N55 (nitrate:ammonium = 5 mM: 5 mM), and N91 (nitrate:ammonium = 9 mM: 1 mM). Each value represents the mean ± SD (*n* = 3). The mean values marked with different letters significantly differ at *P* < 0.05

In Fig. 2, only nitrate significantly promoted the activities of carbon and nitrogen metabolism enzymes, and the activities of Rubisco, SS, NR and GOGAT in *Bn* increased significantly with the increase of nitrate. By contrast, bicarbonate and nitrate/ammonium were not jointd to affect the carbon and nitrogen metabolism enzyme activities, and the bicarbonate insignificantly changed the activities of Rubisco, SS, NRand GOGAT in *Bn*.

Bicarbonate and nitrate were not jointd to affect the carbon and nitrogen metabolism enzymatic activities in *Ov*, and the activities of Rubisco, SS, NR and GOGAT were insignificantly changed at B10N91. The bicarbonate and ammonium joint inhibited the nitrogen metabolism enzymatic activities of *Ov*, and the activities of NR and GOGAT were significantly reduced at B10N19.

### Glucose metabolism

**Fig. 3:**
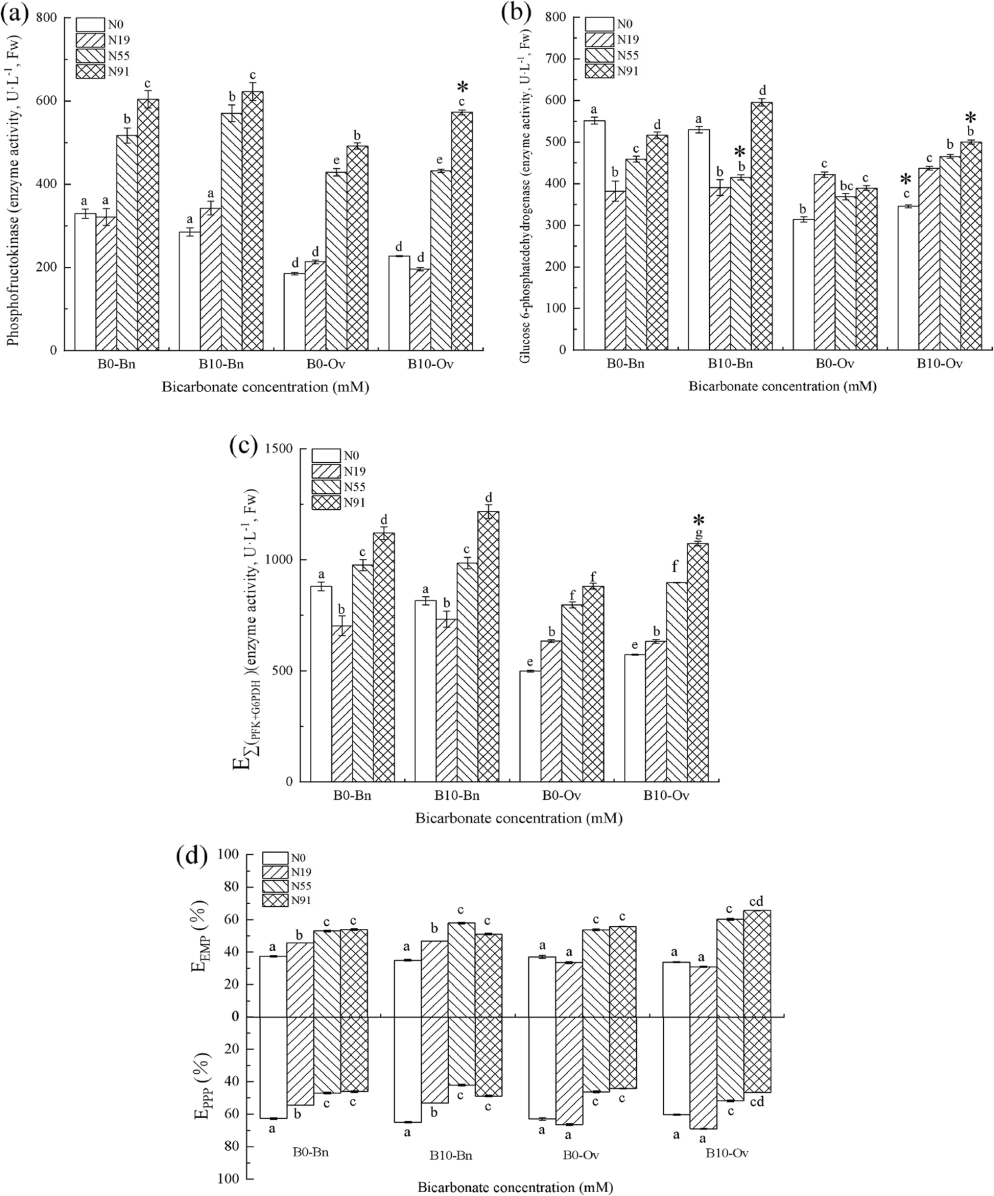
Glycolysis and pentose phosphate pathways of the *Bn* and *Ov* seedlings under simulated karst habitats. *Bn*, *Brassica napus*; *Ov*, *Orychophragmus violaceus*. The bicarbonate concentration denoted as B0 (HCO_3_^−^: 0 mM), and B10(HCO_3_^−^:10 mM). Nitrogen treatments were denoted as N0 (no nitrogen), N19 (nitrate:ammonium = 1 mM: 9 mM), N55 (nitrate:ammonium = 5 mM: 5 mM), and N91 (nitrate:ammonium = 9 mM: 1 mM). Each value represents the mean ± SD (*n* = 3). The mean values marked with different letters significantly differ at *P* < 0.05

In Fig. 3, only nitrate significantly promoted the glucose metabolism in *Bn*. With the increase of nitrate, E_EMP_ and the total glucose metabolic activity significantly increased, while bicarbonate and nitrate/ammonium were not joint to affect the glucose metabolism, and bicarbonate insignificantly changed E_EMP_, E_PPP_ and the total glucose metabolic activity in *Bn*.

The bicarbonate and nitrate joint promoted the glucose metabolism in *Ov*, with the total glucose metabolic activity significantly increasing at B10N91. The bicarbonate and ammonium were not joint to affect the glucose metabolism, without significant changes in E_EMP_, E_PPP,_ and the total glucose metabolic activity at B10N19 in *Ov*.

### The growth capacity and the regeneration capacity of RUBP

**Fig. 4:**
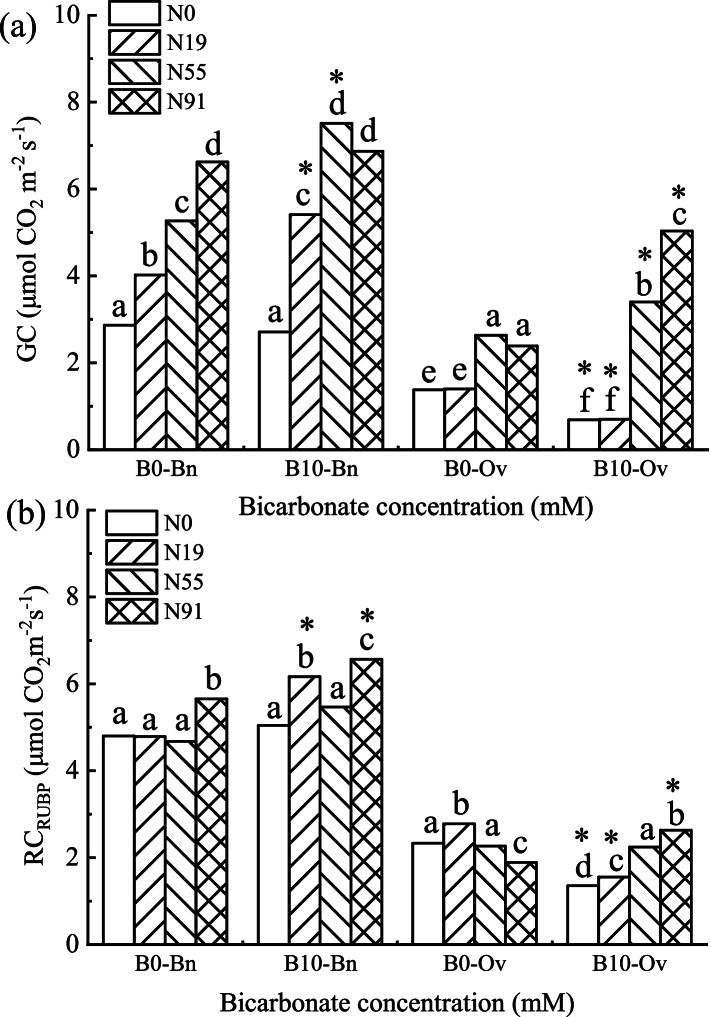
Growth capacity and regeneration capacity of RUBP in *Bn* and *Ov* under simulated karst habitats. *Bn*, *Brassica napus*; *Ov*, *Orychophragmus violaceus*. The bicarbonate was denoted as B0 (HCO_3_^−^: 0 mM), and B10 (HCO_3_^−^:10 mM). Nitrogen treatments were denoted as N0 (no nitrogen), N19 (nitrate:ammonium = 1 mM: 9 mM), N55 (nitrate:ammonium = 5 mM: 5 mM), and N91 (nitrate:ammonium = 9 mM: 1 mM). Each value represents the mean ± SD (*n* = 3). The mean values marked with different letters significantly differ at *P* < 0.05

As shown in Fig. 4, only nitrate significantly promoted the growth capacity (GC) and the regeneration capacity of RUBP (RC_RUBP_) in *Bn*. With the increase of nitrate, GC and RC_RUBP_ were promoted significantly, while bicarbonate and nitrate joint promoted the RC_RUBP_ of *Bn*, which increased significantly at B10N91. However, bicarbonate and ammonium had no significant joint effect on GC and RC_RUBP_ in *Bn*.

The bicarbonate and nitrate joint promoted GC in *Ov*, with a significant increase at B10N91, while the bicarbonate and ammonium joint inhibited it, with a significant decrease at B10N19.

### Leaf carbon and nitrogen content in *Bn* and *Ov*

**Fig. 5:**
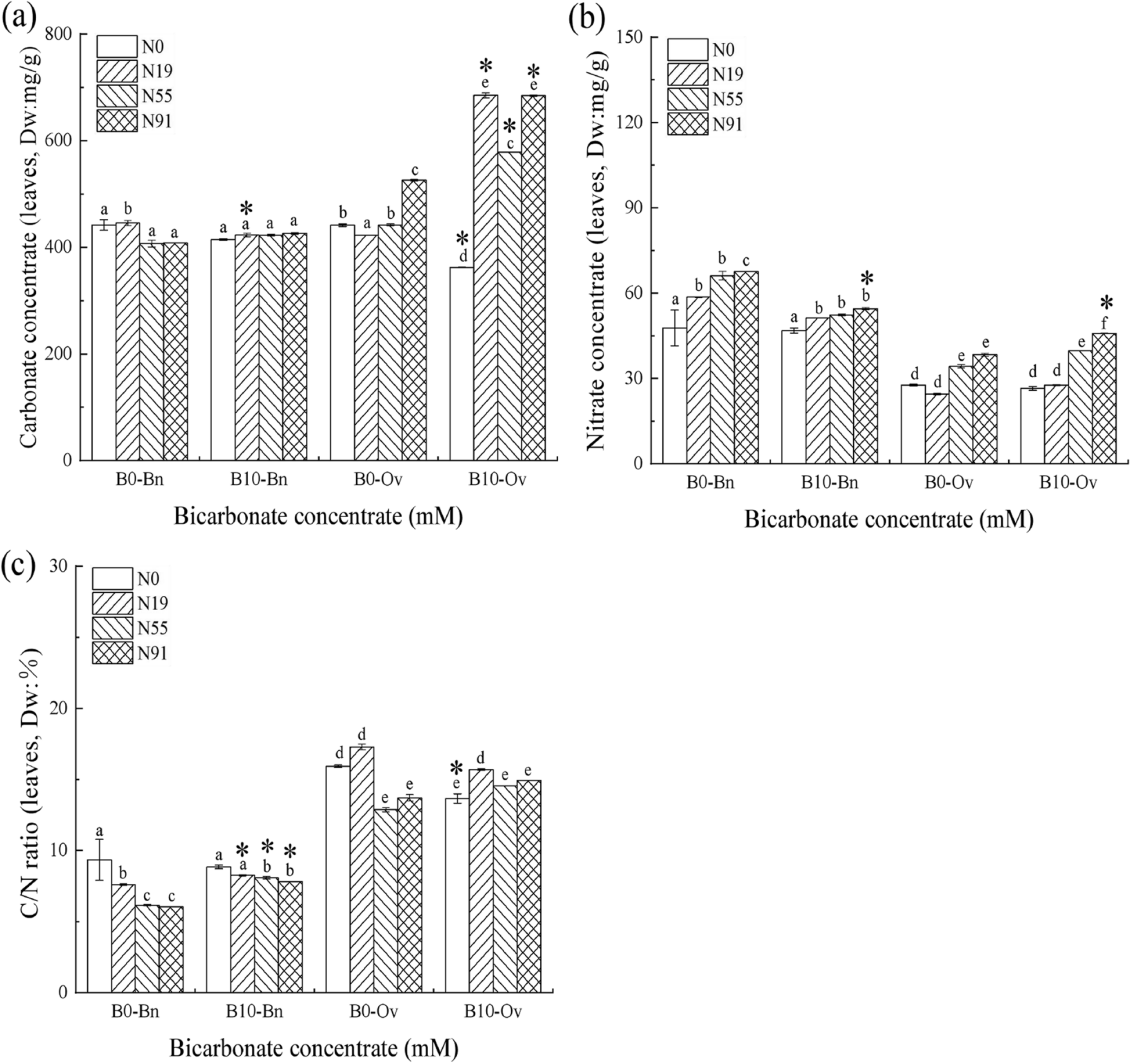
Carbon content, nitrogen content, and C/N ratio of the *Bn* and *Ov* under simulated karst habitats; *Bn*, *Brassica napus*; *Ov*, *Orychophragmus violaceus*. The bicarbonate was denoted as B0 (HCO_3_^−^: 0 mM), and B10 (HCO_3_^−^:10 mM). Nitrogen treatments were denoted as N0 (no nitrogen), N19 (nitrate:ammonium = 1 mM: 9 mM), N55 (nitrate:ammonium = 5 mM: 5 mM), and N91 (nitrate:ammonium = 9 mM: 1 mM). Each value represents the mean ± SD (*n* = 3). The mean values marked with different letters significantly differ at *P* < 0.05

As shown in Fig. 5, only nitrate significantly promoted the leaf nitrogen content in *Bn*, which increased significantly at B0N91. In contrast, bicarbonate and nitrate/ammonium had no significant joint effect on the leaf carbon and nitrogen content in *Bn*.

The bicarbonate and nitrate joint promoted the leaf carbon/nitrogen content in *Ov*, and the leaf carbon/nitrogen content as well as C/N ratio increased significantly at B10N91, while bicarbonate and ammonium had no significant joint effects on the leaf carbon/nitrogen content in *Ov*, the leaf carbon /nitrogen content and C/N ratio did not change insignificantly at B10N19 in *Ov*.

### Biomass

As shown in Table 2, the bicarbonate and nitrate joint promoted the roots, stems, leaves and total biomass in *Bn* and *Ov*, and they all increased significantly at B0N91. Compared to *Bn*, *Ov* had more significant total biomass. Bicarbonate and ammonium were not joint to affect the total biomass in either *Bn* or *Ov*, but the total biomass of B10N91 was higher than that of B0N19.

**Table 2:** Biomass of *Bn* and *O*v under different bicarbonate and nitrogen in simulated karst habitat

Group	HCO_3_^−^(mM)	B0				B10			
Nitrate (mM)	Group	N0	N19	N55	N91	N0	N19	N55	N91
Root (mg)	*Bn*	117.39 ± 1.93a	91.83 ± 1.16a	177.75 ± 1.62b	261.61 ± 3.08c	84.06 ± 1.26a	180.55 ± 25.05b	231.13 ± 2.27c	322.53 ± 5.26d
	*Ov*	40.21 ± 2.28a	44.18 ± 3.78a	58.88 ± 2.17b	51.42 ± 1.4c	40.1 ± 2.09a	75.71 ± 2.75d	121.99 ± 4.91e	344.52 ± 7.29f
Stem (mg)	*Bn*	346.77 ± 7.83a	435.74 ± 11.2 ab	767.17 ± 1.57c	1002.09 ± 2.09d	242.25 ± 13.73e	430.93 ± 13.29ab	1015.82 ± 6.77f	1269.75 ± 29.99 g
	*Ov*	50.5 ± 7.46a	63.65 ± 2.36b	66.9 ± 2.45b	49.82 ± 1.28a	53.6 ± 1.89a	78.74 ± 1.67b	161.01 ± 6.48c	320.65 ± 6.79d
Leaves (mg)	*Bn*	286.43 ± 14.88a	448.65 ± 5.33b	520.41 ± 2.85bc	788.23 ± 7.34d	220.59 ± 11.21a	543.09 ± 18.51bc	671.89 ± 4.44c	1145.25 ± 34.92e
	*Ov*	64.96 ± 4.30a	76.59 ± 11.06ab	130.36 ± 6.51c	93.94 ± 2.42b	105.27 ± 3.97bc	121.13 ± 2.2c	276.06 ± 11.1d	456.85 ± 9.67e
Total biomass (mg)	*Bn*	750.59 ± 9.99a	976.23 ± 11.75b	1465.33 ± 4.43c	2051.93 ± 7.03d	546.9 ± 18.45e	1154.58 ± 32.38b	1918.84 ± 4.99d	2737.53 ± 36.09f
	*Ov*	155.39 ± 11.07a	197.34 ± 33.92b	256.78 ± 11.24c	195.53 ± 5.04b	200.87 ± 5.04b	273.22 ± 9.94c	559.07 ± 22.49d	1122.01 ± 23.75e
root/shoot ratio (R/S, %)	*Bn*	18.55 ± 0.62a	10.39 ± 0.15b	13.81 ± 0.31c	14.61 ± 0.25c	18.16 ± 0.15d	18.54 ± 1.34d	13.7 ± 0.19c	13.35 ± 0.05c
	*Ov*	25.87 ± 1.35a	22.9 ± 1.83a	22.99 ± 0.31a	26.35 ± 0.23ab	25.23 ± 0.41ab	37.87 ± 0.8c	27.91 ± 0b	44.31 ± 0d

Legends: *Bn*, *Brassica napus*; *Ov*, *Orychophragmus violaceus*. The bicarbonate was denoted as B0 (HCO_3_^−^: 0 mM), and B10 (HCO_3_^−^:10 mM). Nitrogen treatments were denoted as N0 (no nitrogen), N19 (nitrate:ammonium = 1 mM: 9 mM), N55(nitrate:ammonium = 5 mM: 5 mM), and N91 (nitrate:ammonium = 9 mM: 1 mM). Each value represents the mean ± SD (*n* = 3). The mean values marked with different letters significantly differ at *P* < 0.05

## Discussion

### Joint interactions of bicarbonate and nitrate

#### Joint promotions on bicarbonate and nitrate in *Bn* and *Ov*

In this study, we found that nitrate enhanced the carbon and nitrogen metabolisms in both *Ov* and *Bn.* This is because nitrate promoted nitrate reduction and facilitated enzyme formation, then enhanced the Rubisco, SS, NR and GOGAT activities that promoted the carbon and nitrogen metabolisms in plants [25, 26].

The joint promotion of bicarbonate and nitrate in *Bn* and *Ov* was different, and the bicarbonate and nitrate joint promoted the photosynthesis, glucose metabolism and growth only in *Ov.* Bicarbonate provided protons for nitrate reduction, which promoted the formation of NADPH and RC_RUBP_, resulting in the enhancement of the photosynthesis and biomass in plants [27]. However, the promotion in *Bn* was offset by the adverse effect of bicarbonate [28, 29]. Compared to *Bn*, *Ov* was more capable of utilizing bicarbonate. On the one hand, it decreased the toxicity of bicarbonate [1]. On the other hand, the carbonic anhydrase of *Ov* was increased, resulting in more HCO_3_^−^ catalyzed into H_2_O and CO_2_ to alleviate the stomatal closure and water use efficiency under karst drought, thus promoting the recovery of photosynthesis [1, 2]. Finally, the total glucose metabolism and biomass in *Ov* were promoted (Fig. 1).

#### Joint inhibitions on bicarbonate and nitrate in *Bn* and *Ov*

In this study, we found that the bicarbonate and nitrate joint only inhibited Tr of *Ov*. Compared to *Bn*, *Ov* was more capable of utilizing bicarbonate under karst drought [1, 2]. Dominated to CA, which excellently catalyzed HCO_3_^−^ to CO_2_ and H_2_O [4, 5], thus increasing the water use efficiency, and reducing Tr in *Ov* (Fig. 1).

### Joint interactions of bicarbonate and ammonium

#### Joint promotions on bicarbonate and ammonium in *Bn* and *Ov*

In this study, the bicarbonate and ammonium joint only promoted Ci in *Bn* and WUE in *Ov*. Compared to *Ov*, *Bn* had a greater demand for ammonium [30, 31], which made nitrogen accumulation increase dramatically, leading the photosynthesis enhance [32]. Therefore, the demand for CO_2_ and Ci increased in *Bn*. Meanwhile, *Ov* had lower demand for ammonium and therefore the nitrogen assimilation decreased, leading to the decrease of photosynthesis and leaf area [33]. Consequently, the transpiration decreased and the water use efficiency significantly enhanced in *Ov*.

#### Joint inhibitions on bicarbonate and ammonium in *Bn* and *Ov*

In this study, the bicarbonate and ammonium joint inhibited both the photosynthesis and the nitrogen metabolism in *Ov*. Previous studies have shown that bicarbonate can provide electrons to balance the cell potential imbalance caused by extra ammonium in the alkaline environment, thus reducing intracellular acidification [34, 35]. Therefore, the *Bn* enhanced its leaf biomass by using more ammonium, which significantly promoted Tr, resulting in the decrease of WUE in *Bn*. Compared to *Bn*, the *Ov* consumed a lower amount of ammonium, which decreased the nitrogen accumulation, resulting in the decline of the C and N metabolism enzymes, photosynthesis, NR., GOGAT, Pn and GC in *Ov* (Figs. 1 and 2).

#### Joint interactions of C and N in karst-adaptable plants

**Fig. 6:**
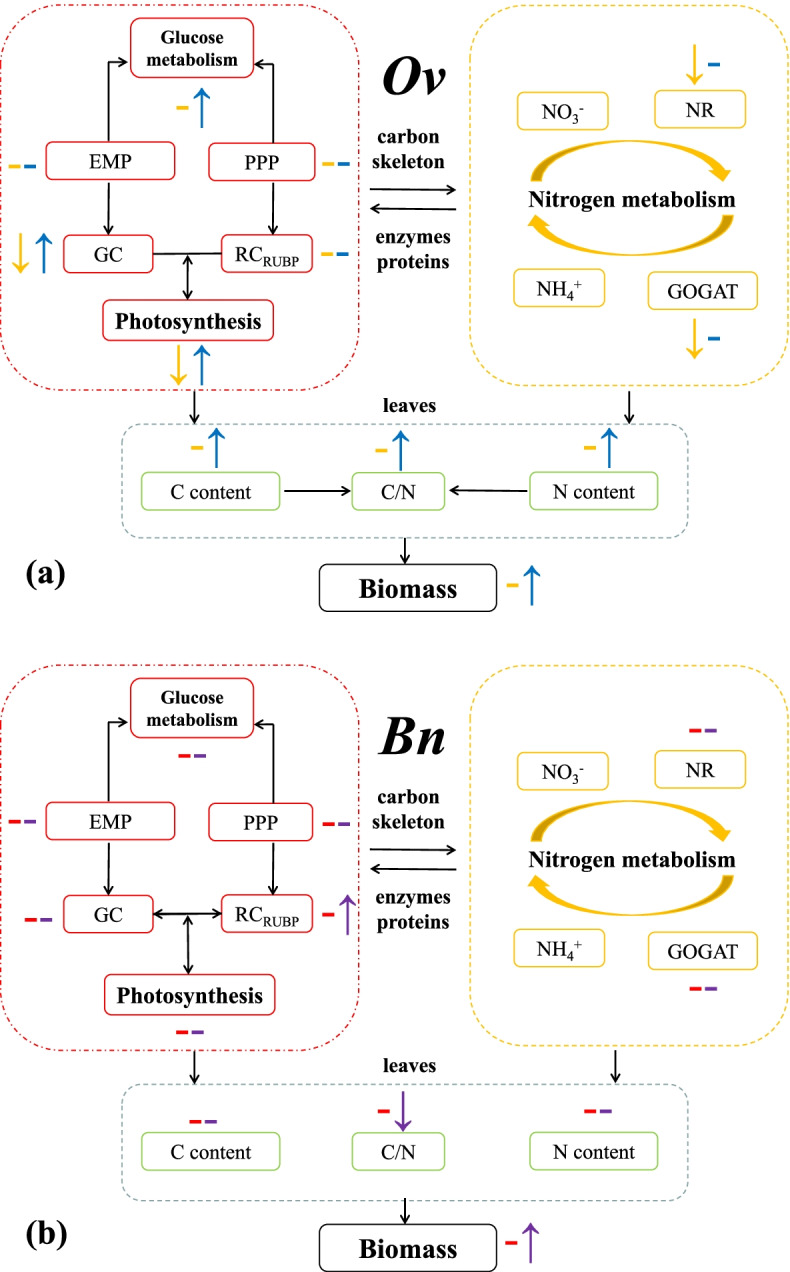
The carbon and nitrogen joint effects in *Bn* and *Ov* under simulated karst habitats. ↑: joint promotion; −: no significant conjugation; ↓: joint inhibition; *Ov*: the yellow line indicates the joint interactions of bicarbonate and ammonium, and the blue line indicates the joint interactions of bicarbonate and ammonium; *Bn*: the red line indicates the joint interactions of bicarbonate and ammonium, and the purple line indicates the joint interactions of bicarbonate and nitrate

The *Bn* and *Ov* have different carbon/nitrogen coupling mechanisms under karst habitats (Tables 3 and 4), and the latter is more adaptable to high bicarbonate, high nitrate and high pH [1, 2, 5]. In this study, we found that the bicarbonate and nitrate joint promoted the photosynthesis, glucose metabolism and water use efficiency in *Ov*, enabling it to adapt well to the drought, high bicarbonate, abundant nitrate and bare aluminium habitats. At the same time, the bicarbonate and ammonium joint inhibited the carbon/ nitrogen metabolism and growth of *Ov*, but promoted the water use efficiency, helping *Ov* alleviate the poison of ammonium to resistant the karst habitats (Fig. 6a). Additionally, in this study, bicarbonate and nitrate/ammonium did not clearly joint affected the carbon/nitrogen metabolism in *Bn* (Fig. 6b), attributing to its weak karst adaptions. Hence, the C and N joint interactions are vital physiological mechanisms of karst adaptations in *Ov*.

**Table 3:** The carbon/nitrogen joint interactions in *Bn* and *Ov* under karst habitats

Group	Conjugation	*Bn*	*Ov*
HCO_3_^−^ × NO_3_^−^	Promotion	Cond, RC_RUBP_, Biomass	Pn, Cond, WUE, PFK, G6PDH, E_∑_, Biomass
	Inhibition	–	Tr
HCO_3_^−^ × NH_4_^+^	Promotion	Ci	WUE
	Inhibition	–	Pn, Cond, Tr, Ci, NR, GOGAT, GC

**Table 4:** Adaptive habitats of different metabolisms in *Bn* and *Ov*

Metabolism process	Karst drought		High bicarbonate and high nitrate		High bicarbonate and low ammonium	
	*Ov*	*Bn*	*Ov*	*Bn*	*Ov*	*Bn*
Photosynthesis	**+**	**–**	**+**	**–**	**+**	**–**
NR/GOGAT	**+**	**–**	**+**	**–**	**+**	**–**
Glucose metabolism	**+**	**–**	**+**	**–**	**+**	**–**
Carbon/nitrogen accumulation	**+**	**–**	**+**	**–**	**+**	**–**

Legends: +: adaptation; −: non adaptation

## Conclusions

The bicarbonate and nitrate/ammonium jointly affected the carbon and nitrogen metabolisms, of which there were significant differences between a karst-adaptable plant (*Ov*) and a non-karst-adaptable plant (*Bn*). The *Ov* was more adaptable to karst habitats as bicarbonate and nitrate joint promoted photosynthesis and glucose metabolism in *Ov*, enhancing the carbon and nitrogen metabolism and growth, while they didn’t significantly affect the carbon and nitrogen metabolism in *Bn.* Bicarbonate and ammonium joint inhibited the photosynthesis and nitrogen metabolism in *Ov*. Also, they promoted water use efficiency, leading the growth to delay, and the ammonium toxicity alleviation, as well as the resistance to drought enhance, but the *Bn* was barely affected*.* In general, the joint interactions of carbon and nitrogen metabolisms in karst adaptable plants, such as *Ov* and others, are more sensitive to bicarbonate and nitrate/ammonia, which is essential for their adaptions to karst habitats. Furthermore, clarifying the joint interactions of various metabolisms, which dominated to the growth of plant species, also takes great contributions to the sustainable development of karst area.

## Supplementary Information

**Additional file 1: Table S1.** The original experimental data in this study.

## Data Availability

Data generated or analyzed during this study were included in this published article and its supplementary information files.

## Abbreviations

Bn: *Brassica napus*
Ci: Intercellular CO_2_ concentration
Cond: Stomatal conductivity
EMP: Glycolytic pathway
GC: Growth capacity
G6PDH: Glucose-6-phosphate dehydrogenase
GOGAT: Glutamate synthase
HCO_3_^−^: Bicarbonate
NH_4_^+^: Ammonium
NO_3_^−^: Nitrate
NR: Nitrate reductase
Ov: *Orychophragmus violaceus*
PFK: Phosphofructokinase
Pn: Photosynthetic rate
PPP: Pentose phosphate pathway
RC_RUBP_: The regeneration capacityof RUBP
Rubisco: Ribulose bisphosphate carboxylase oxygenase
SS: Sucrose synthetase
Tr: Transpiration
WUE: Water use efficiency
